# A Novel Reading Scheme for Assessing the Extent of Radiographic Abnormalities and Its Association with Disease Severity in Sputum Smear-Positive Tuberculosis: An Observational Study in Hyderabad/India

**DOI:** 10.1371/journal.pone.0138070

**Published:** 2015-09-18

**Authors:** Zarko Grozdanovic, Luis C. Berrocal Almanza, Surabhi Goyal, Abid Hussain, Tilman E. Klassert, Dominik Driesch, Viktoriya Tokaryeva, Yvonne Yi-Na Löschmann, Gadamm Sumanlatha, Niyaz Ahmed, Vijayalakshmi Valluri, Ralf R. Schumann, Birgit Lala, Hortense Slevogt

**Affiliations:** 1 Department of Radiology, Campus Benjamin Franklin, Charité - Universitätsmedizin Berlin, Berlin, Germany; 2 Institute for Microbiology and Hygiene, Campus Mitte, Charité - Universitätsmedizin Berlin, Berlin, Germany; 3 Septomics Research Center, Jena University Hospital, Jena, Germany; 4 Department of Immunology, Bhagwan Mahavir Medical Research Centre, Hyderabad, India; 5 BioControl Jena GmbH, Jena, Germany; 6 Department of Radiology, Helios Klinikum Berlin-Buch, Berlin, Germany; 7 Pathogen Biology Laboratory, Department of Biotechnology and Bioinformatics, University of Hyderabad, Hyderabad, India; 8 Immunology and Molecular Biology, LEPRA Society- Blue Peter Research Centre, Hyderabad, India; 9 Department of Pediatric Radiology, Campus Virchow-Klinikum, Charité - Universitätsmedizin Berlin, Germany; National Institute of Infectious Diseases, JAPAN

## Abstract

**Background:**

Existing reading schemes for chest X-ray (CXR) used to grade the extent of disease severity at diagnosis in patients with pulmonary tuberculosis (PTB) are often based on numerical scores that summate specific radiographic features. However, since PTB is known to exhibit a wide heterogeneity in pathology, certain features might be differentially associated with clinical parameters of disease severity.

**Objective:**

We aimed to grade disease severity in PTB patients at diagnosis and after completion of DOTS treatment by developing a reading scheme based on five different radiographic manifestations and analyze their association with the clinical parameters of systemic involvement and infectivity.

**Methods:**

141 HIV-negative adults with newly diagnosed sputum smear-positive PTB were enrolled in a prospective observational study in Hyderabad, India. The presence and extent on CXRs of five radiographic manifestations, i.e., lung involvement, alveolar infiltration, cavitation, lymphadenopathy and pleural effusion, were classified using the new reading scheme by using a four-quadrant approach. We evaluated the inter-reader reliability of each manifestation, and its association with BMI and sputum smear positivity at diagnosis. The presence and extent of these radiographic manifestations were further compared with CXRs on completion of DOTS treatment.

**Results:**

At diagnosis, an average lung area of 51.7% +/- 23.3% was affected by radiographic abnormalities. 94% of the patients had alveolar infiltrates, with 89.4% located in the upper quadrants, suggesting post primary PTB and in 34.8% of patients cavities were found. We further showed that the extent of affected lung area was a negative predictor of BMI (β value -0.035, p 0.019). No significant association of BMI with any of the other CXR features was found. The extent of alveolar infiltrates, along with the presence of cavitation, were strongly associated with sputum smear positivity. The microbiological cure rate in our cohort after 6 months of DOTS treatment was 95%. The extent of the affected lung area in these patients decreased from 56.0% +/- 21.5% to 31.0 +/- 20% and a decrease was also observed in the extent of alveolar infiltrates from 98.4% to 25.8% in at least one quadrant, presence of cavities from 34.8% to 1.6%, lymphadenopathy from 46.8% to 16.1%, and pleural effusion from 19.4% to 6.5%.

**Conclusions:**

We established a new assessment scheme for grading disease severity in PTB by specifically considering five radiographic manifestations which were differently associated with the BMI and sputum smear positivity, changed to a different extent after 6 months of treatment and exhibited an excellent agreement between radiologists. Our results suggest that this reading scheme might contribute to the estimation of disease severity with respect to differences in disease pathology. Further studies are needed to determine a correlation with short and long-term pulmonary function impairment and whether there would be any benefit in lengthening or modulating therapy based on this CXR severity assessment.

## Introduction

Tuberculosis (TB) remains a global public health problem with an estimated 9.0 million incident cases and 1.5 million deaths in 2013 [1]. India alone accounts for an estimated 2.2 million cases [2]. According to the WHO guidelines for TB control, early diagnosis of pulmonary TB (PTB) by smear microscopy remains the main procedure in low-income settings, even though it has the drawbacks of low specificity and variable sensitivity [3]. In India, the Revised National Tuberculosis Control Program RNTCP recommends the use of chest radiography (CXR), where available, as an additional screening tool to increase the sensitivity of the diagnostic algorithm [4]. In this context, certain radiographic features and scoring systems have been described for the identification of PTB by CXR [5]. However, these features are not fully pathognomonic for PTB and lack reproducibility, especially when used in the field [5–8].

CXR also potentially provides useful information for grading the extent of pulmonary involvement and disease severity at diagnosis, as well as for assessing treatment outcome [9, 10]. The pathologic features of PTB exhibit wide heterogeneity [10]. In this context, e.g., in reports from the pre-antibiotic era, at least two distinct types of pathology in pulmonary TB—a nodular form and tuberculous pneumonia—have been described [11]. Therefore, it seems likely that specific radiographic features at diagnosis, as well as after treatment, might differ between patients not only as a result of disease severity, but also differences in disease pathology [10, 11]. The extent of infected lung tissue destruction is linked to disease severity, bacillary load, and disease spread [11]. In addition, lung remodeling and pathological scarring often occurs during the course of the disease, despite pathogen clearance under Directly Observed Treatment Short-course (DOTS) treatment [12–16]. As a result, short- and long-term disease effects can lead to persistent impairment of lung function, despite microbiological cure, in a significant proportion of patients [13, 17]. To analyze whether specific radiographic features are related to disease severity at diagnosis, they can be correlated with other well-known parameters of disease severity and infectivity, such as BMI, sputum smear positivity and the FEV1. Some of the CXR features have already been found to be related to the extent of pulmonary pathology and are associated with clinical signs of disease severity and infectivity: BMI is a well-recognized clinical measure of TB severity [18–20]; and malnutrition is also known to be associated with the radiographic extent of pulmonary disease [14, 20–22]. Several studies have also shown that patients with cavitary PTB have higher bacterial loads in their sputum [23–26]. Moreover, in one study, the area of alveolar infiltrates was also found to be associated with sputum smear positivity [18].

However, these studies used different CXR reading schemes for the characterization of pulmonary involvement, e.g., by dividing the lungs into parts: upper and lower lobes [23, 26], thirds [13, 18], quarters [27], by using additional scores [13, 27] or by grading the density of infiltrates [20, 27]. Consequently, a new numerical scoring system was suggested to grade disease severity at diagnosis and predict treatment outcome by using two-month smear status as an outcome measure [22]. This score was derived from the percentage of affected lung by grading visual estimation of the extent of any dense or patchy opacification, cavitation, or other pathology as percentage plus 40, if cavitation was present [22]. These authors also demonstrated that this numerical score correlated with BMI, sputum smear positivity, as well as FEV1, as well-known clinical parameters of disease severity [22]. However, the expression of pulmonary involvement as a numerical score might not reflect the heterogeneity of pulmonary pathology since different CXR features are not related to disease severity to the same extent. For instance, areas of higher density on CXR due to pulmonary scaring which also contribute to the extent of the area of lung affected might not mirror active disease and thus the extent of tissue destruction to the same degree than alveolar infiltrates which are most likely reflecting the early inflammatory state of acute post primary PTB and in particular of tuberculous pneumonia [11].

We hypothesized that distinct CXR features are differentially associated with disease severity and might thus be differently associated to the BMI and sputum smear positivity as clinical parameters of disease severity. In addition it is not known whether and to what extent specific CXR features at diagnosis are associated with lung impairment after the completion of treatment. Therefore to independently grade different CXR features that have been associated with PTB might allow an improved description of CXR changes in TB patients after DOTS treatment in the field, which might help to facilitate more tailor-made treatment in TB care, in general [24, 28]. The aim of this study was to grade disease severity in HIV-negative adult patients with newly diagnosed sputum smear-positive PTB, in a prospective study in Hyderabad/India, using a new system that independently determines the presence and extent of five different manifestations of radiographic findings that are associated with PTB [18, 24, 28, 29]. The following radiographic manifestations were classified: (i) extent of lung involvement, (ii) extent of alveolar infiltration, (iii) the presence of cavitation, (iv) the presence of lymphadenopathy, and (v) the presence of pleural effusion. With regard to the manifestation `extent of lung involvement´, we combined and simplified the reading schemes for this manifestation suggested by Ralph et al. [22], and the radiographic reading scheme suggested by Baez-Saldana et al. [27] making use of a quadrant scheme. In addition, the presence or absence of cavities, mediastinal and/or hilar lymphadenopathy and pleural effusion was noted, but the extent of these manifestations was not assessed. In order to justify the assumption that each manifestation of our CXR reading scheme correlates with other well-known parameters of disease severity at diagnosis, we analyzed its association with the clinical parameters of systemic involvement (BMI) and infectivity (sputum smear positivity). The presence and extent of the radiographic manifestations on CXR at diagnosis were further compared with those following the completion of 6 months of DOTS treatment, to further assess whether changes in the appearance of the different CXR manifestations with treatment were associated with subsequent improvement.

## Materials and Methods

### Study design, setting, and participants

We performed an observational study at the Mahavir hospital and research center in Hyderabad, India. The recruitment of study participants was carried out prospectively from July 26, 2011 to November 21, 2013; all 141 individuals with PTB within the study period were recruited according to the following inclusion criteria. We recruited only patients who were newly diagnosed with a sputum smear-positive PTB disease, who did not have previous exposure to TB antibiotic therapy and were registered in the Hyderabad DOTS program at Mahavir hospital. All participants tested negative for HIV. The diagnostic criterion for the presence of PTB was the presence of at least two initial sputum smear examinations positive for Acid-Fast Bacillus (AFB), in accordance with the Indian technical and operational guidelines for TB control [30]. The DOTS treatment was according to the RNTCP and consisted of one intensive phase with isoniazid (H), rifampicin (R), pyrazinamide (Z), and ethambutol (E) administered under direct observation three times per week on alternate days, for 2 months, followed by a continuation phase for 4 months with H and R administered thrice per week on alternate days with at least the first dose of every week being directly observed [31, 32]. The dosage strengths of drugs used under DOTS consist of H: (600 mg), R: (450 mg), Z: (1500 mg), E: (1200 mg) or S (streptomycin): (750 mg). Patients who weighed 60 kg or more received additional 150 mg R, patients who were more than 50 years old received 500 mg S instead of E, patients who weighed less than 30 kg received drugs according to body weight [31]. All study participants gave their written informed consent before the investigations and the study was approved by the institutional ethics committee for bio-medical research, Bhagwan Mahavir Medical Research Centre, Hyderabad, India.

### Variables, data sources and measurements

Demographic data, including gender, age and BMI, along with data on smoking and drinking habits, were recorded using the hospital’s standard DOTS program questionnaire. The final treatment outcome was recorded as cured, failed, default, lost to follow-up, and death. The demographic data were obtained directly from patients or their relatives during an interview performed by a trained researcher at initial recruitment to the study. Other relevant clinical data, including the results of the sputum smear microscopy and the serological HIV test result, were obtained from the hospital’s TB control program records and medical staff. Sputum smears were examined at the time of diagnosis and after two month of treatment for AFB using Ziehl–Neelsen staining and graded by standard criteria as follows: no AFB in 100 High-Power Field (HPF) (negative), 1–9 AFB in 100 HPF (Scanty), 10–99 AFB in 100 HPF (1+), 1–10 AFB per field in at least 50 HPF (2+) and > 10 AFB per HPF in at least 20 (3+) [33].

### Assessment of the chest radiographs

All patients underwent postero-anterior CXR at the start of DOTS treatment using a Siemens Heliophos D X-ray generator (Siemens, Mumbai-India). Because CXR is not part of the standard follow-up for PTB patients at Mahavir hospital’s DOTS program, only 62 patients also underwent additional CXR after completing 6 months of treatment. CXRs were first assessed by two radiologists who, by consensus, developed an assessment scheme for the radiographic manifestations indicative of the presence and extent of lung involvement in PTB, as described below. Subsequently, two independent radiologists analyzed these manifestations to assess inter-reader reliability. All radiologists were blind in relation to the clinical data. In order to define the extent of the two pulmonary manifestations area of lung involvement and alveolar infiltrates, each lung was divided into two halves, an upper and a lower half, by a horizontal line drawn at the level of the upper endplate of a thoracic vertebra situated immediately below the tracheal carina. The four resulting quadrants were designated as follows: I, right upper quadrant (RUQ), II, right lower quadrant (RLQ), III, left upper quadrant (LUQ), and IV, left lower quadrant (LLQ) S1 Fig, and as proposed by Baez-Saldana et al. [27]. The total area of lung affected by any parenchymal lesion was determined by counting the number of quadrants involved, i.e., from one to four and each quadrant accounting for 25% of the total extent. This is a more simplified scheme than the one recently proposed which is based on the total percentage of affected lung by visual estimation of the extent and density of opacification, cavitation, or other pathology as a percentage of visible lung [22]. In a next step, the extent of alveolar infiltrates was separately graded using the four-quadrant scheme. Alveolar infiltrate, a term still preferably used by clinicians, relates to areas of patchy or confluent opacities, in contrast to nodular lesions, airway disease, a miliary pattern, or parenchymal scarring. Moreover, cavities, when present, were assigned to one or more quadrants, e.g., I for cavitation in RUQ. No attempt was made to evaluate the size of the cavities, and the presence or absence of mediastinal and/or hilar lymphadenopathy along with pleural effusion was noted, but the extent or localization in the four-quadrant scheme of these manifestations was not assessed.

### Statistical methods

Continuous variables were expressed as mean ± Standard Deviation (SD) or median ± Interquartile Range (IQR) and categorical variables as the frequency count and percentage. Inter-observer agreement between radiologists regarding categorical variables was assessed with kappa statistics, the initial kappa values were prevalence and bias, adjusted as previously described [34], and interpreted according to the approach of Landis et al. [35]. The agreement in continuous variables was assessed using correlation analysis and the method of Bland and Altman [36]. The association of individual CXR features and clinical characteristics was assessed with univariable and multivariable regression analysis: linear regression was used for continuous dependent variables, such as the BMI, and ordinal regression for ordinal dependent variables such as the sputum smear microscopy. The goodness of fit of the regression models was evaluated with ANOVA and goodness of fit tests for linear and ordinal regressions respectively, and the amount of variation explained by the model with the R^2^ and pseudo R^2^ tests for linear and ordinal regressions, respectively. In the case of ordinal regression, the proportional odds assumption was assessed with the test of parallel lines. The differences between the extent of CXR features at diagnosis and after completion of 6 months of DOTS treatment were analysed with the Wilcoxon Signed Rank test, McNemar test, and repeated measures multinomial regression for continuous, categorical binary and categorical ordinal variables, respectively. The difference in BMI gain, depending on the affected lung area, was assessed with the Kruskal-Wallis test. SPSS 21 (SPSS Inc., Chicago, Illinois-USA) was used for data analysis and values of *p* <0.05 were considered significant. The Strengthening the Reporting of Observational Studies in Epidemiology (STROBE) Guidelines were used as a reporting guide for this observational study [37].

## Results

### Socio-demographic and clinical characteristics of study participants

We carried out a survey in 141 newly diagnosed PTB patients whose main socio-demographic and clinical characteristics are shown in Table 1. This was a relatively young population 26.9 ± 11.4 years with an almost equal number of male 50.4% and female 49.6% participants. AFB sputum smear microscopy showed that 58.1% of the patients had a bacterial burden of 2+ or 3+, 33.3% had 1+ and 8.5% had a scanty burden. Most of the patients 93.6% had negative sputum results after two months of DOTS treatment Table 1. The result for the final treatment outcome in the 141 recruited patients was that 138 patients completed the 6 months treatment: one patient defaulted and did not come back to the hospital, one patient died prematurely shortly after starting therapy and another was transferred to another hospital. Two out of 138 patients who completed treatment were diagnosed with MDR and two patients who had been previously reported as cured returned to the hospital following relapse.

**Table 1:** Characteristics of study participants.

Variable	n = 141
Age mean (SD), (years)	26.9 ± 11.4
Gender n (%), male/female	71 (50.4)/70 (49.6)
BMI mean (SD), (Kg/m^2^)	16.3 ± 2.5
Smoking mean (SD), pack-year	0.20 ± 0.81
Drinking n (%), (yes/no)	27 (19.1)/114 (80.9)
Sputum smear grade at diagnosis n (%)	
Scanty	12 (8.5)
1+	47 (33.3)
2+	35 (24.8)
3+	47 (33.3)
2 months sputum smear status n (%)	
Negative	132 (93.6)
Positive	9 (6.4)
6 months outcome n (%)	
Cured	134 (95)
Failed	4 (2.8)
Default	1 (0.7)
Transferred	1 (0.7)
Died	1 (0.7)

(SD) standard deviation.

### Chest radiography (CXR) findings

The CXR for each patient after being enrolled in the study was evaluated. On average, 54.7% ± 23.3% of lung area was affected Table 2. The CXR revealed that 94.3% of patients had alveolar infiltrates, with different levels ranging from the first to the fourth lung quadrant; in addition, 41.8% of patients had lymphadenopathy. Cavitation was present in 29.1% of patients. In a next step, we assessed the presence of primary and post-primary PTB by dividing the patients into two groups, according to the presence of alveolar infiltrates in the upper or lower quadrants of their CXRs, and found that 10.6% had lower-lobe infiltrate and 89.4% had upper-lobe infiltrate Table 3, which suggests that most of the patients in our cohort were most likely affected by post-primary PTB, according to conventional criteria [28].

**Table 2:** Specific Chest Radiograph (CXR) features of lung involvement.

Variable	n = 141
**Alveolar infiltrates** n (%)	
None	8 (5.7)
1 quadrant	41 (29.1)
2 quadrants	61 (43.3)
3 quadrants	21 (14.9)
4 quadrants	10 (7.1)
**Lymphadenopathy** n (%), (yes/No)	59 (41.8)/82 (58.2)
**Cavitation** n (%)	
None	100 (70.9)
1 Cavity	34 (24.1)
2 Cavities	7 (5)
**Affected lung area** mean (SD)	54.7 ± 23.3
**Pleural effusion** n (%), (yes/No)	23 (16.3)/118 (83.7)

(SD) standard deviation.

**Table 3:** Chest Radiograph (CXR) results according to the presence of alveolar infiltrates in the upper or lower lung lobe.

Variable	n = 141	Lower lobe infiltrate	Upper lobe infiltrate
**Alveolar infiltrates** n (%)		15 (10.6)	126 (89.4)
None	8 (5.7)	7 (46.7)	1 (0.8)
1 quadrant	41 (29.1)	7 (46.7)	34 (27)
2 quadrants	61 (43.3)	1 (6.7)	60 (47.6)
3 quadrants	21 (14.9)		21 (16.7)
4 quadrants	10 (7.1)		10 (7.9)

### Assessment of the inter-reader reliability of the CXR features according to our assessment scheme

The results of the inter-reader reliability evaluation for the identification of the CXRs manifestations, i.e., (i) affected lung area, (ii) alveolar infiltrates, as well as the presence of (iii) cavitation, (iv) pleural effusion, and (v) lymphadenopathy are summarized in Table 4. Our results show a significant agreement among the radiologists in identifying the extent of alveolar infiltrates. In addition, there was a high level of agreement 73% in identifying the affected lung area. Pleural effusions were also identified with a high level of agreement. However, only a moderate level of agreement was found when determining the presence of cavitation and only a fair level of agreement when identifying the presence of lymphadenopathy. All kappa values were prevalence-adjusted and bias-adjusted, a procedure that improved the level of agreement. As an example, S2A, S2B, S2C and S2D Fig show CXRs with respective agreement or disagreement among radiologists in interpreting these lesions as cavities.

**Table 4:** Inter-rater agreement on Chest Radiograph (CXR) findings.

**Inter-rater agreement for categorical variables**	**Kappa**	**P value**	**Prevalence and bias adjusted kappa**	**Interpretation of prevalence and bias adjusted kappa**
Alveolar infiltrates	0.421	0.005	0.720	Substantial
Lymphadenopathy	0.285	0.060	0.348	Fair
Cavitation	0.480	<0.0001	0.510	Moderate
Pleural effusion	0.448	0.002	0.636	Substantial
**Agreement for continuous variables**		***r***_***c***_	**P value**	**95% limits of agreement (Bland and Altman)**
Affected lung area		0.734	<0.0001	-50 to 31.4%

### The affected lung area is negatively associated with the BMI

Consequently, in order to assess whether individual CXR manifestations could be used as correlates of disease severity, we analyzed their association with BMI, as an indicator of disease severity, and the degree of sputum smear positivity as an indicator of infectivity [20, 24]. Initially, we employed BMI as dependent variable to test which CXR feature could be a predictor of this parameter with linear regression analysis. The initial univariable analysis revealed that the extent of alveolar infiltrates and the affected lung area are negative predictors of BMI (β value -0.620, p 0.008) and (β value -0.034, p <0.0001), (Table 5, models 1 and 2, respectively); however, when the two variables were analyzed together in a multivariable model, only the extent of affected lung area became a negative predictor of BMI (β value -0.035, p 0.019), (Table 5, model 3). No significant association of BMI with any of the other CXR features was found.

**Table 5:** Univariable linear regression: association of affected lung area with BMI

Predictor	β	S.E	P value	95%CI for β
**Model 1**Alveolar infiltrates	- 0.620	0.229	0.008	-1.073–-0.167
**Model 2**Affected lung area	- 0.034	0.009	<0.0001	-0.052–-0.016
**Model 3**Alveolar infiltrates	0.044	0.359	0.903	-0.666–0.753
Affected lung area	- 0.035	0.015	0.019	-0.065–-0.006

**Model parameters dependent variable BMI: Model 1** Constant 17.53 ANOVA 0.008, R^2^ 0.044. **Model 2** Constant 18.22 ANOVA <0.0001, R^2^ 0.087. **Model 3** Constant 18.21 ANOVA 0.002, R^2^ 0.076.

### The extent of alveolar infiltrates and the presence of cavitation are associated with the degree of sputum smear positivity

As a next step, we assessed the association of the presence and extent of all CXR manifestations with the degree of sputum smear positivity at baseline. We first evaluated the association of each CXR manifestation by univariable analysis, followed by multivariable analysis, which included only those CXR manifestations with significant associations in univariable analysis. For this purpose, we used sputum smear positivity as an ordinal categorical variable, for which there is a clear order within the different categories of the variable. Accordingly, in addition to being able to classify a TB patient as sputum smear- positive or negative, the patient could be assigned to one of the four smear categories: scanty, 1+, 2+ and 3+; e.g., a patient in a higher category would have more bacteria in the sputum than those in the category below. In the univariable analysis, we found that the extent of alveolar infiltrates, the presence of cavities, and the affected lung area were associated with the extent of sputum smear positivity (Table 6, model 1, 2 and 3, respectively). However, the multivariable analysis revealed that only the extent of alveolar infiltrates and the presence of cavities were independent predictors of sputum smear positivity (Table 6, model 4).

**Table 6:** Univariable and multivariable ordinal regression: association of alveolar infiltrates and cavities with number of bacteria in sputum smear.

Predictor	β	S.E	P value	Exp β (OR)	95%CI for Exp β (OR)
**Model 1**Alveolar infiltrates					
1 quadrant	1.395	0.736	0.058	4.04	0.95–17.06
2 quadrants	1.573	0.719	0.029	4.82	1.82–19.76
3 quadrants	2.436	0.803	0.002	11.43	2.37–55.21
4 quadrants	2.659	0.923	0.004	14.29	2.34–87.21
**Model 2**Cavitation					
No cavities	-1.760	0.845	0.037	0.17	0.03–0.90
1 cavity	-1.471	0.881	0.095	0.23	0.04–1.29
**Model 3**Affected lung area	0.016	0.007	0.019	1.02	1.00–1.03
**Model 4**					
Alveolar infiltrates					
1 quadrant	1.345	0.743	0.070	3.84	0.90–16.45
2 quadrants	1.465	0.772	0.058	4.33	0.95–19.63
3 quadrants	2.382	0.952	0.012	10.83	1.68–70.01
4 quadrants	2.552	1.221	0.037	12.83	1.17–140.5
Cavitation					
No cavities	-1.727	0.874	0.048	0.18	0.03–0.99
1 cavity	-1.605	0.914	0.079	0.20	0.03–1.21
Affected lung area	0.001	0.012	0.997	1.00	0.98–1.02
**Model 5**					
Alveolar infiltrates					
1 quadrant	1.345	0.742	0.070	3.84	0.90–16.44
2 quadrants	1.465	0.727	0.044	4.33	1.04–19.99
3 quadrants	2.383	0.813	0.003	10.84	2.20–53.84
4 quadrants	2.553	0.945	0.007	12.84	2.02–81.77
Cavitation					
No cavities	-1.727	0.871	0.047	0.18	0.03–0.98
1 cavity	-1.605	0.907	0.077	0.20	0.03–1.19

**Dependent ordinal variable sputum smear (<1+, 1+, 2+ and 3+). Model 1** -2Log 52, Chi^2^ 0.015, Goodness of fit 0.88, Pseudo R^2^ (Cox and snell 0.084 and Nagelkerke 0.091), test of parallel lines 0.021. **Model 2** -2Log 27.5, Chi^2^ 0.068, Goodness of fit 0.98, Pseudo R^2^ (Cox and snell 0.037 and Nagelkerke 0.040), test of parallel lines 0.971. **Model 3** -2Log 53, Chi^2^ 0,020, Goodness of fit 0,083, Pseudo R^2^ (Cox and snell 0,067 and Nagelkerke 0,041), test of parallel lines 0,240. **Model 4** -2Log 158, Chi^2^ 0.08, Goodness of fit 0,012, Pseudo R^2^ (Cox and snell 0.11 and Nagelkerke 0.12), test of parallel lines 0.022. **Model 5** -2Log 100, Chi^2^ 0.009, Goodness of fit 0,080, Pseudo R^2^ (Cox and snell 0.11 and Nagelkerke 0.12), test of parallel lines 0.075. **Reference categories:** Sputum smear: 3+, Alveolar infiltrates: No infiltration, Cavities: 2 cavities.

We performed a further multivariable analysis including only the extent of alveolar infiltrates and the presence of cavities. This new multivariable model showed that patients with alveolar infiltrates in two quadrants of the lungs had a higher probability of having the value 3+ in their sputum smear compared to those with no infiltration (Odds Ratio (OR) 4.33, 95%CI 1.04–19.99) (p 0.044) (Table 6, model 5). The results were similar for patients with alveolar infiltrates in three and four quadrants of the lungs, (OR 10.84, 95%CI 2.20–53.84) (p 0.003) and (OR 12.84, 95%CI 2.02–81.77) (p 0.007), respectively (Table 6 model 5). The association in patients with alveolar infiltrates in only the first quadrant did not reach statistical significance (OR 3.84, 95%CI 0.90–16.44) (p 0.070). However, the results of the other categories suggest a trend towards having 3+ in the sputum smear being more likely with an increasing number of quadrants with alveolar infiltrates. Similarly, patients with no cavities in the lungs were less likely to have the value 3+ in their sputum smear compared to those with two cavities (OR 0.18, 95%CI 0.03–0.98), (p 0.047), while the difference between patients with one cavity and two cavities did not reach statistical significance (Table 6, model 5). These results indicate that the extent of alveolar infiltrates and the presence of cavities are associated with the grade of sputum smear positivity.

### There are differences in drinking habits depending on the extent of alveolar infiltrates and presence of cavitation

We stratified the study population by the extent of alveolar infiltrates and cavitation, in order to illustrate how drinking and smoking habits, which have been associated with an increased risk for PTB, and other clinical and CXR features are distributed according to these two manifestations of pulmonary involvement. This analysis revealed that patients with alveolar infiltrates in all four lung quadrants on CXR reported a higher frequency of drinking habits 60% (p 0.018). Moreover, patients with two lung cavities also reported a higher frequency of drinking habits 57.1%, (p 0.009) S1 Table, however, the difference in smoking habits according to levels of infiltration and cavitation did not reach statistical significance S1 Table. These results suggest that there are differences in drinking but not smoking habits related to the extent of alveolar infiltrates and the presence of cavities.

### The proportion of affected lung area and cavitation do not predict neither two-month sputum smear status nor treatment outcome

A scoring system for grading CXR severity in adults with smear-positive PTB based on the proportion of total lung area affected plus 40, if cavitation was present, for predicting the two-month sputum smear status was recently reported [22]. We assessed whether the proportion of affected lung area and cavitation, as defined and assessed in our reading scheme, are predictors of two-month sputum smear status. We performed regression analysis to assess the association of the proportion of affected lung area and cavitation with two-month sputum smear status in two separate univariable models and, subsequently, the association with two-month sputum smear status when the two variables were combined into a score, as recently suggested [22]. However, in our study population and using our CXR reading scheme, neither cavitation nor the affected lung area separately, or combined into a score, were associated with two-month sputum smear results (OR 0.48, 95%CI -0.046–-0.004) (p 0.30), (OR 1.00, 95%CI 0.978–1.036), (p 0.63) and (OR 1.01, 95%CI 0.994–1.037), (p 0.16), (Table 7 models 1, 2 and 3 respectively). Next, we evaluated whether there are differences in the proportion of affected lung area and cavitation between patients that were cured and those who were not cured after six months DOTS treatment, this analysis did not reveal any significant differences S2 Table.

**Table 7:** Univariable logistic regression: association of CXR score based on the affected area of the lung and presence of cavitation with two-month sputum smear.

Predictor	β	S.E	P value	Exp β (OR)	95%CI for Exp β
**Model 1**Cavitation	- 0.720	0.698	0.303	0.487	-0.046–-0.004
**Model 2**Affected lung area	0.007	0.015	0.637	1.007	0.978–1.036
**Model 3**CXR score: affected lung area plus 40 if cavitation present	0.015	0.011	0.167	1.015	0.994–1.037

**Model parameters: Model 1** constant:- 2.22, -2Log likelihood 65.9-Chi^2^ 0.31, Hosmer and lemeshow test <0.0001, R^2^ (Cox 0.007 and Nagelkerke 0.019). **Model 2** constant:- 3.07, -2Log likelihood 66.7-Chi^2^ 6.37, Hosmer and lemeshow test 0.206, R^2^ (Cox 0.002 and Nagelkerke 0.004). **Model 3** constant:- 3.77, -2Log likelihood 65-Chi^2^ 1.67, Hosmer and lemeshow test 0.446, R^2^ (Cox 0.013 and Nagelkerke 0.036).

### Changes in the extent of CXR manifestations after 6 months of DOTS treatment

In our study, the microbiological cure rate after completion of the treatment was 95%, although 6.4% of patients still had a positive sputum smear after two months of treatment Table 1. To assess whether the specific CXR manifestations showed differential improvements after treatment, we performed a repeated measures analysis in 62 patients who also had CXR after completion of DOTS treatment. As illustrated in Table 8, after 6 months of DOTS treatment the extent of affected lung area decreased from 56.0% +/- 21.5% to 31.0% +/- 20%. A decrease was also observed in the extent of alveolar infiltrates in at least one quadrant from 98.4% to 25.8%, presence of cavities from 34.8% to 1.6%, lymphadenopathy from 46.8% to 16.1%, and pleural effusion from 19.4% to 6.5%. These data suggest that after 6 months of treatment and despite a significant overall improvement in CXR pulmonary involvement, PTB patients still showed radiographic abnormalities in their lungs. Subsequently, to assess whether the severity of lung involvement was associated with less BMI gain after 6 months of DOTS treatment, we compared the BMI gain between patients according to their affected lung area. This analysis revealed no difference in BMI gain dependent on affected lung area S3 Fig

**Table 8:** Changes in CXR specific features at diagnosis and after 6 months of DOTS therapy.

Variable	At diagnosisn = 62	6 months therapyn = 62	P value
**Alveolar infiltrates** n (%)			
No	2 (3.2)	46 (74.4)	
1 quadrant	16 (25.8)	13 (21)	
2 quadrants	32 (51.6)	2 (3.2)	<0.0001
3 quadrants	7 (11.3)	1 (1.6)	
4 quadrants	5 (8.1)		
**Lymphadenopathy** n (%), (yes/No)	29 (46.8)/33 (53.2)	10 (16.1)/52 (83.9)	<0.0001
**Cavitation** n (%)			
No	46 (74.2)	61 (98.4)	
1 Cavity	15 (24.8)	1 (1.6)	0.002
2 Cavities	1 (1.6)		
**Affected lung area** mean (SD)	56 ± 21.5	31 ± 20	<0.0001
**Pleural effusion** n (%), (yes/No)	12 (19.4)/50 (80.6)	4 (6.5)/58 (93.5)	0.008

## Discussion

In this study, our aim was to grade the severity of pulmonary involvement of PTB with a new reading scheme based on the assessment of the presence and extent of different radiographic manifestations associated with PTB [22, 27]. The extent of lung involvement and the extent of alveolar infiltrates were graded by making use of a four quadrant scheme. The presence of cavities, lymphadenopathy and pleural effusion were included as either present or not present. By applying this radiographic reading scheme, we found that, in our PTB patients, an average lung area of 51.7% +/- 23.3% was affected by radiographic abnormalities at diagnosis. In addition, 94% of the patients had alveolar infiltrates at least in one quadrant and, in 89.4% of the cases, infiltration could be observed in the upper quadrants, suggesting that most of these patients had post primary PTB. This is in agreement with other studies which reported that post primary TB occurs in immunocompetent adults and accounts for about 80% of all clinical cases and nearly 100% of infection transmission [29, 38]. In our study, cavitation was found in 29.1% of the patients, which is in agreement with the prevalence reported by other studies in PTB patients [39]. Interestingly, good inter-reader reliability could be found for grading the affected lung area, as well as for grading the extent of alveolar infiltrates, suggesting that our simple reading scheme has the potential for accurate and reproducible CXR grading of disease severity of PTB. For the detection of cavities, inter-reader reliability was surprisingly moderate. Therefore, cavitation can be a very distinct feature of PTB that is easy to detect, but in other settings, it can be overlooked [18, 39].

To substantiate our finding that different CXR manifestations correlate with BMI and sputum smear positivity as adequate well-known parameters of disease severity at diagnosis, we found that the extent of lung involvement was related to BMI, whereas the extent of alveolar infiltrates, along with the presence of cavitation, was strongly associated with sputum smear positivity. Our findings are in agreement with those that found a similar correlation between BMI and radiographic features of lung involvement [20]. By assessing the extent of alveolar infiltrates with the new reading scheme, we found that this feature was associated with sputum smear positivity. In the early inflammatory state of acute post primary PTB, the extent of local pulmonary disease pathology, and in particular of tuberculous pneumonia, has been shown to be associated with the bacillary load in the sputum of TB patients [28, 32, 40]. Therefore, our findings support the observation that the extent of alveolar infiltrates assessed with the new reading scheme reflects this early state in PTB and its relation to increased infectivity. As expected, we also found an association between sputum smear positivity and the presence of cavities, which is in agreement with the findings of several other studies [25, 41, 42]. The presence of lymphadenopathy and pleural effusion, albeit with a fair level of agreement, was neither associated with BMI nor with sputum smear positivity. Consequently, these CXR features most likely reflect the local disease status.

Our results suggest that the use of individual CXR manifestations might be more appropriate and practical in assessing disease severity than the use of a score system. Although the extent of alveolar infiltrates and cavitation are both predictors of sputum smear positivity, alveolar infiltration has a higher level of agreement between radiologists and, therefore, it might be a more reliable proxy marker of infectivity. It is also probable that each CXR manifestation reflects different pathophysiological stages of the disease. We judged the association of the proportion of affected lung area and cavitation as evaluated in our reading scheme with two-month sputum smear status as recently suggested [22], but we did not find an association between the extents of affected lung area or cavitation and two-month sputum smear status. However, the methodology that we used to assess lung involvement was not entirely comparable to the one previously used [22], which might be the reason for the discrepancies between the two studies. A recent meta-analysis revealed that sputum smear examination at the end of the second month of treatment has only low sensitivity and modest specificity for predicting treatment failure and relapse, therefore it might not greatly add to scoring systems for predicting the outcome of PTB [43].

The microbiological cure rate in our study after 6 months of DOTS treatment was 95%, although 4% of patients still had positive sputum smear after two months of treatment. Accordingly, CXR findings of all patients improved after 6 months of treatment. But, approximately one third of the patients still showed PTB associated radiographic abnormalities in at least one quadrant. Noteworthy, it was recently shown that in patients with cured PTB, the degree of radiographic abnormalities evaluated using a scoring system comparable to the one used in our study, was significantly and inversely associated with spirometric impairment assessed by FVC and FEV1 [27]. These findings have recently been confirmed by another study demonstrating that 34.3% of patients with a history of PTB had non-reversible chronic airway obstruction that was associated with significantly more radiographic abnormalities in CXRs [44]. It is therefore likely that, despite the microscopically cure rate of 95%, the existing pulmonary lesions in the CXRs of our patients might at least partly reflect persistent respiratory impairments. Since CXR is often not available for the diagnosis and follow up of PTB patients in low income settings, there are only few data comparing the radiographic pulmonary involvement at diagnosis and after 6 months of DOTS treatment. A recent study reported that after 6 months of DOTS treatment, 27% of TB patients still had at least moderate-severe pulmonary function impairment, and 57% still had respiratory symptoms, despite most achieving 'successful' treatment outcomes [45]. These findings support an association of the area of lung affected with severity of pulmonary involvement. The extent of alveolar infiltrates as a different and more distinct manifestation of our reading scheme decreased from 98.4% to 25.8% of patients with infiltrates in at least one quadrant. It is likely that a considerable proportion of the patients analyzed in a previous study in which a reduced FEV1 could be related to the area of lung affected, was due to the presence of persistent alveolar infiltrates [27]. However, in follow-up studies, this manifestation was not independently investigated in terms of its association with PTB pathology and its impact on long-term outcome so far.

One additional finding of our study was that, after 6 months of treatment, the presence of cavities decreased substantially from 34,8% to 1.6%, which was an excellent cure rate compared to another study which found that from 53.1% of the PTB patients who presented cavities in CXR at diagnosis, 16.9% of subjects still had persistent cavities at the end of 6 months of treatment, that was associated with an increased risk of TB relapse [46]. Further studies are needed in order to investigate which factors are associated with the reduction in the presence of cavities observed in our study. In our cohort, we also found that lymphadenopathy decreased from 46.8% to 16.1% and pleural effusion from 19.4% to 6.5% after 6 months of treatment. No data are available that associate lymphadenopathy independently with long-term outcome or short- and long-term pulmonary function impairment. But, in one study, persistent pleural effusion in PTB was shown to be associated with pleural thickening and a slowed FEV1 and FVC increase after 6 months treatment [47].

Taken together, we found that most of the radiological manifestations we investigated were differentially associated with parameters of disease severity and each changed to a different extent after 6 months treatment. Since, according to the RNTCP, the standard DOTS treatment regime for all PTB patients was based on a three times per week application, it is tempting to speculate that patients with severe disease, according to different manifestations, might benefit from an intensification of the frequency of antibiotic therapy. One of the limitations of our study was the absence of other clinical parameters correlated with disease severity, such as underlying lung disease, as well as FEV1, which would have allowed the assessment of its association with individual CXR manifestations and their confounding effect on the association between the extent of CXR features with BMI and sputum smear grade. In addition, only 62 patients could be followed by CXR from diagnosis to the completion of DOTS treatment and, since we only studied CXRs from HIV-negative smear positive patients, our findings need to be evaluated further for HIV-positive and/ or smear-negative patients with PTB.

In conclusion, we have developed a new assessment scheme to grade the presence and extent of specific CXR features and pulmonary disease severity in patients with PTB. By differentially analyzing the five radiographic manifestations, i.e., lung involvement, alveolar infiltration, cavitation, lymphadenopathy and pleural effusion our grading scheme factors the broad heterogeneity in pulmonary pathology allowing the integration of differential information regarding disease severity into clinical decision-making. Prospective studies are required to evaluate this reading scheme in representative populations, and to determine the association of each manifestation with short- and long-term pulmonary function impairment and whether there would be any benefit to lengthening or modulating therapy based on CXR severity assessment.

## Supporting Information

S1 Fig Sketch showing the division of each lung into two parts by a horizontal line used to determine the area of lung affected. (ZIP)

S2 Fig Chest radiographs of four patients with newly diagnosed smear positive TB. In each radiograph a lesion is marked by an arrow. There was either agreement (a, b) or no agreement (c, d) among radiologists in interpreting these lesions as cavities.
(ZIP)

S3 Fig There is no difference in weight gain between patients according to the area of lung affected. The BMI gain in (kg/m^2^) after 6 months DOTS treatment was calculated by subtracting the BMI at 0 months to the BMI at 6 months and was compared between patients according to the area of lung affected. Data are represented as the median ± interquartile range (IQR) and statistical difference was assessed with Kruskal-Wallis test, n = 9 in 25%, n = 30 in 50%, n = 14 in 75% and n = 6 in 100%.
(ZIP)

S1 Table Characteristics of study participants stratified by the presence of alveolar infiltrates and cavitation. (DOCX)

S2 Table Comparison of the presence of alveolar infiltrates and cavitation in cured and not cured patients. (DOCX)
