# Supplementary figures and images for: A Novel Reading Scheme for Assessing the Extent of Radiographic Abnormalities and Its Association with Disease Severity in Sputum Smear-Positive Tuberculosis: An Observational Study in Hyderabad/India
Source: PLoS One. 2015 Sep 18;10(9):e0138070. doi: 10.1371/journal.pone.0138070 (PMC4575099; doi:10.1371/journal.pone.0138070)

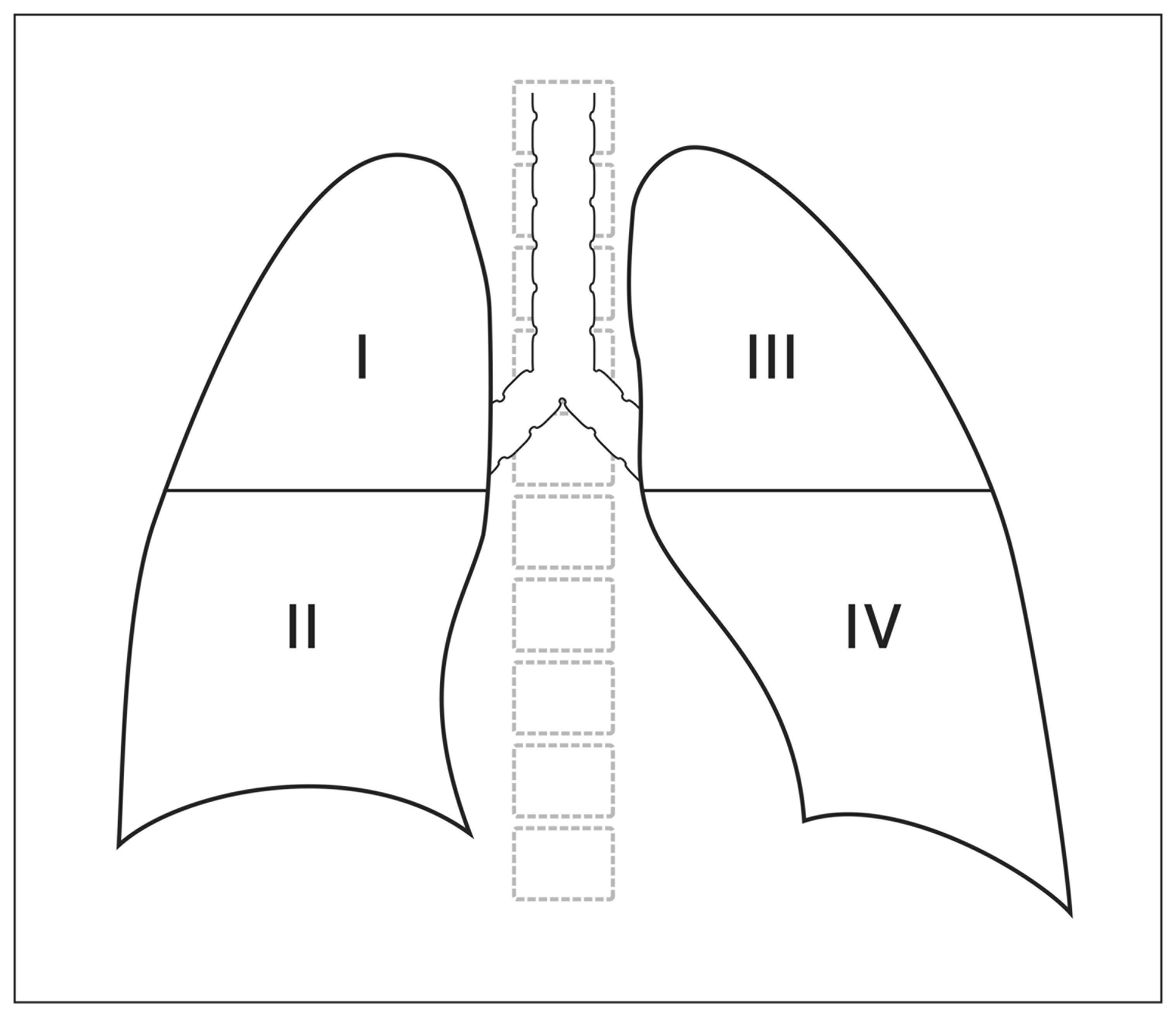

Supplement: S1 Fig — (ZIP) [file pone.0138070.s001.zip › S1 Figure.tif]

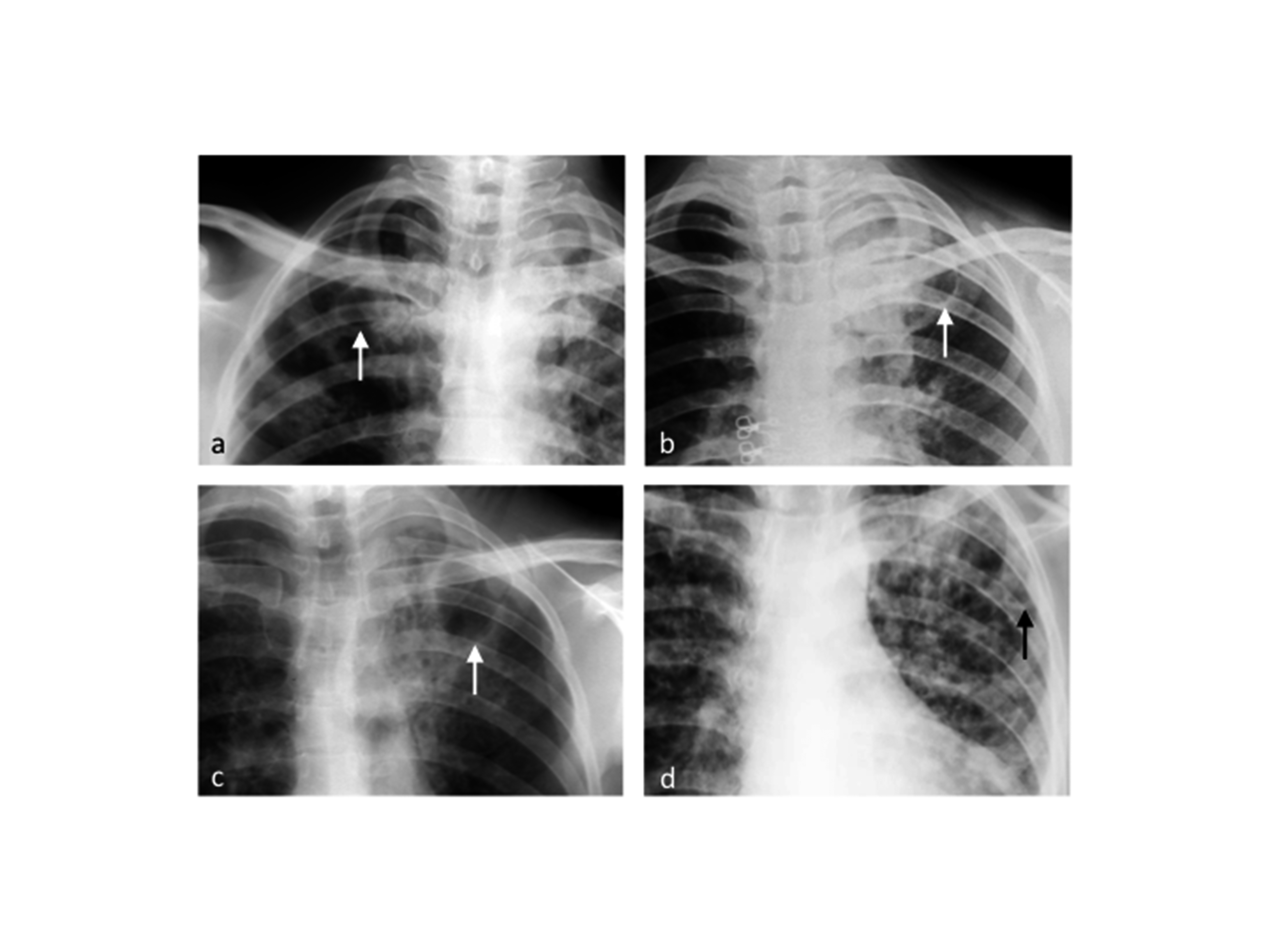

Supplement: S2 Fig — In each radiograph a lesion is marked by an arrow. There was either agreement (a, b) or no agreement (c, d) among radiologists in interpreting these lesions as cavities. (ZIP) [file pone.0138070.s002.zip › S2 Figure.tif]

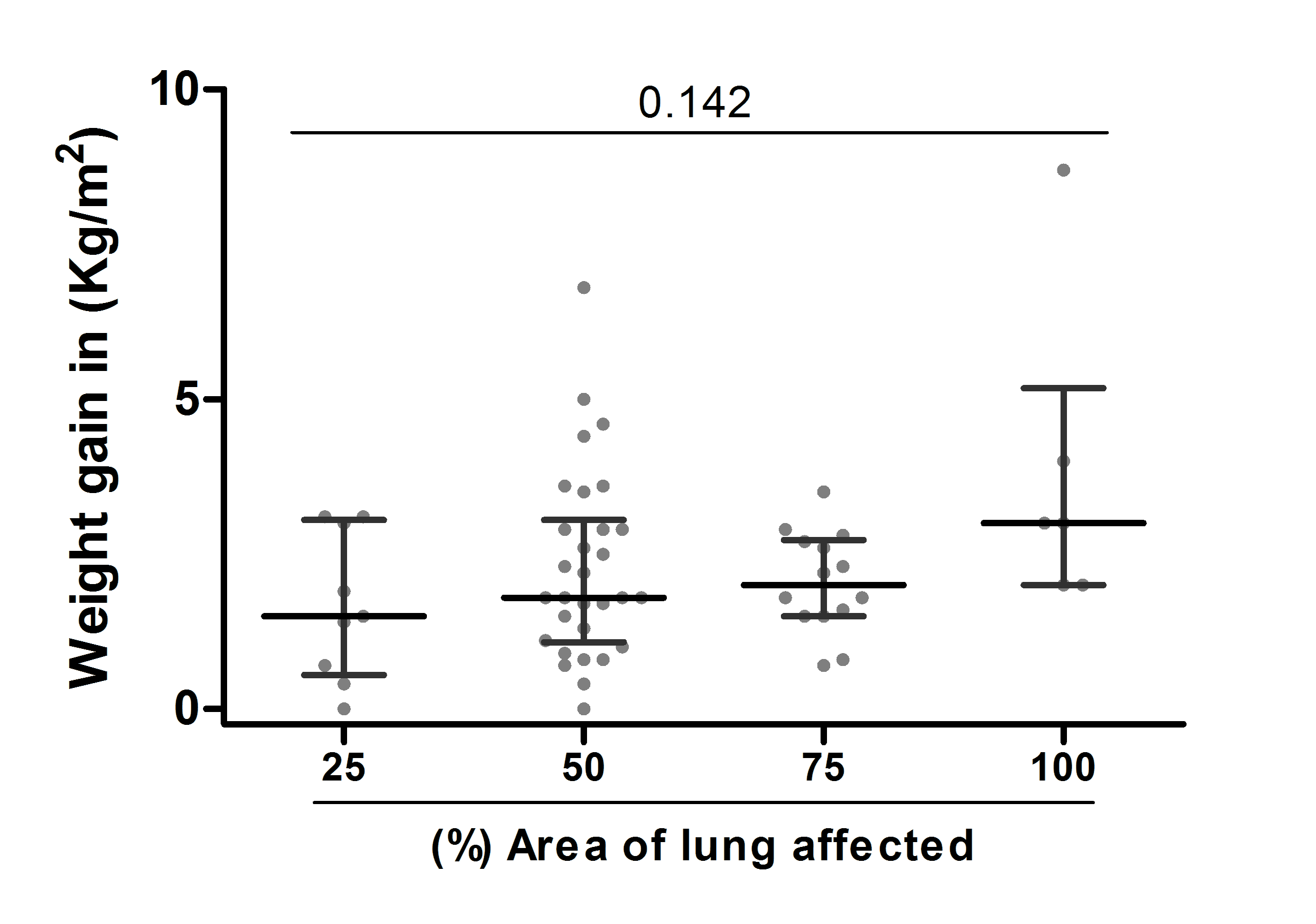

Supplement: S3 Fig — The BMI gain in (kg/m2) after 6 months DOTS treatment was calculated by subtracting the BMI at 0 months to the BMI at 6 months and was compared between patients according to the area of lung affected. Data are represented as the median ± interquartile range (IQR) and statistical difference was assessed with Kruskal-Wallis test, n = 9 in 25%, n = 30 in 50%, n = 14 in 75% and n = 6 in 100%. (ZIP) [file pone.0138070.s003.zip › S4 Figure.tif]
