# Supplementary material for: A Novel Reading Scheme for Assessing the Extent of Radiographic Abnormalities and Its Association with Disease Severity in Sputum Smear-Positive Tuberculosis: An Observational Study in Hyderabad/India
Source: PLoS One. 2015 Sep 18;10(9):e0138070. doi: 10.1371/journal.pone.0138070 (PMC4575099; doi:10.1371/journal.pone.0138070)
Supplement: S1 Table — (DOCX) [file pone.0138070.s004.docx]

**S3 Table:** Characteristics of study participants stratified by the presence of alveolar infiltrates and cavitation.

|  |  | **Alveolar infiltrates** |  |  |  |  | **P value** | **Cavitation** |  |  | **P value** |
| --- | --- | --- | --- | --- | --- | --- | --- | --- | --- | --- | --- |
|  |  | None | 1 quadrant | 2 quadrants | 3 quadrants | 4 quadrants |  | None | 1 cavity | 2 cavities |  |
|  |  | n=8 | n=41 | n=61 | n=21 | n=10 |  | n=100 | n=34 | n=7 |  |
|  | Age median (IQR), (Years) | 24 ± 14.7 | 25 ± 20 | 22 ± 13 | 24 ± 17 | 30 ± 17.5 | 0.911 | 23 ± 17 | 25 ± 15.5 | 38 ± 29 | 0.353 |
|  | Gender n (%), male/female | 3(37.5)/5  (62.5) | 21(51.2)/20  (48.8) | 29(47.5)/32  (52.5) | 11(52.4)/10  (47.6) | 7(70)/3  (30) | 0.672 | 46(46)/54  (54) | 20(58.8)/14  (42.2) | 5(71.4)/2  (28.6) | 0.226 |
|  | Smoking median (IQR), pack-year | 0 | 0.3 ± 1.3 | 0.17 ± 0.53 | 0.1± 0.3 | 0.35± 0.47 | 0.223 | 0.09± 0.35 | 0.45± 1.4 | 0.53±0.73 | 0.058 |
|  | Drinking n (%), (yes/no) | 1(12.5)/7  (87.5) | 6(14.6)/35  (85.4) | 11(18)/30  (82) | 3(14.3)/18  (85.7) | 6(60)/4  (40) | 0.018 | 14(14)/86  (86) | 9(26.6)/  25(73.5) | 4(57.1)/3  (42.9) | 0.009 |
| **Baseline** | BMI median (IQR), (Kg/m^2^) | 15.4 ± 3.5 | 17 ± 3.5 | 16 ± 2.7 | 15.4 ± 2.4 | 13.9 ± 2.2 | 0.004 | 15.8 ± 2.9 | 15.8 ± 3.5 | 15.8 ± 3.6 | 0.561 |
|  | Sputum smear  n (%) |  |  |  |  |  |  |  |  |  |  |
|  | < 1+ | 3 (37.5) | 6 (14.6) | 3 (4.9) |  |  |  | 10 (10) | 2 (5.9) |  |  |
|  | 1+ | 3 (37.5) | 14 (34.1) | 24 (39.3) | 3 (14.3) | 3 (30) | 0.011 | 35 (35) | 11 (32.4) | 1 (14.3) | 0.451 |
|  | 2+ |  | 8 (19.5) | 17 (27.9) | 9 (42.9) | 1 (10) |  | 25 (25) | 9 (26.5) | 1 (14.3) |  |
|  | 3+ | 2 (25) | 13 (31.7) | 17 (27.9) | 9 (42.9) | 6 (60) |  | 30 (30) | 12 (35.3) | 5 (71.4) |  |
|  | **Other CXR features** |  |  |  |  |  |  |  |  |  |  |
|  | Lymphadenopathy n (%), (yes/No) | 3(37.5)/5  (62.5) | 16(39)/25  (61) | 23(37.7)/38  (62.3) | 14(66.7)/7  (33.3) | 3(30)/7  (70) | 0.164 | 46(46)/54  (54) | 10(29.4)/24  (70.6) | 3(42.9)/4  (57.1) | 0.238 |
|  | Affected lung area  median (IQR) | 37.5 ± 43.75 | 25 ± 25 | 50 ± 0 | 75 ± 0 | 100 ± 0 | <0.0001 | 50 ± 25 | 50 ± 50 | 75 ± 25 | 0.283 |
|  | Pleural effusion  n (%), (yes/No) | 0(0)/8  (100) | 4(9.8)/37  (90.2) | 13(21.3)/48  (78.7) | 3(14.3)/18  (85.7) | 3(30)/7  (70) | 0.248 | 19(19)/81  (81) | 3(8.8)/31  (91.2) | 1(14.3)/6  (85.7) | 0.378 |
|  |  |  |  |  |  |  |  |  |  |  |  |
| **2 months** | Sputum smear  n (%) |  |  |  |  |  |  |  |  |  |  |
|  | Positive |  | 2 (4.9) | 3 (4.9) | 4 (19) |  |  | 5 (5) | 2 (5.9) | 2 (28.6) | 0.049 |
|  | Negative | 8 (100) | 39 (95.1) | 58 (95.1) | 17 (81.1) | 100 (100) | 0.124 | 95 (95) | 32 (94.1) | 5 (71.4) |  |
|  |  |  |  |  |  |  |  |  |  |  |  |
| **6 months** | Outcome n (%) |  |  |  |  |  |  |  |  |  |  |
|  | Cured | 8 (100) | 39 (95.1) | 57 (93.4) | 20 (95.2) | 10 (100) |  | 98 (98) | 31 (91.2) | 31 (71.4) |  |
|  | Failed |  | 2 (4.9) | 2 (3.2) |  |  | 0.965 | 2 (2) | 1 (2.9) | 1 (14.3) | <0.0001 |
|  | Default |  |  | 1 (1.6 |  |  |  |  | 1 (2.9) |  |  |
|  | Transferred |  |  | 1 (1.6) |  |  |  |  | 1 (2.9) |  |  |
|  | Died |  |  |  | 1 (4.8) |  |  |  |  | 1 (14.3) |  |

(IQR) interquartile range
