# Supplementary material for: A Novel Reading Scheme for Assessing the Extent of Radiographic Abnormalities and Its Association with Disease Severity in Sputum Smear-Positive Tuberculosis: An Observational Study in Hyderabad/India
Source: PLoS One. 2015 Sep 18;10(9):e0138070. doi: 10.1371/journal.pone.0138070 (PMC4575099; doi:10.1371/journal.pone.0138070)
Supplement: S2 Table — (DOCX) [file pone.0138070.s005.docx]

**S4 Table:** Comparison of the presence of alveolar infiltrates and cavitation in cured and not cured patients.

| Variable | Cured | Not cured | P value |
| --- | --- | --- | --- |
| Cavitation n (%), (yes/no) | 3(60)/2(40) | 34( 25.4)/100(74.6) | 0.117 |
| Affected lung area  median (IQR) | 50 (100) | 50 (37.5) | 0.915 |

(IQR) interquartile range
